# Investigations on the vaginal temperature, cycle stages, and steroid hormone concentrations during the breeding season in camels (*Camelus dromedarius*)

**DOI:** 10.14202/vetworld.2021.1102-1108

**Published:** 2021-05-07

**Authors:** Ragab H. Mohamed, Amal M. Abo El-Maaty, Rasha S. Mohamed, Axel Wehrend, Fatma Ali, Hassan A. Hussein

**Affiliations:** 1 Department of Theriogenology, Faculty of Veterinary Medicine, Aswan University, Aswan, Egypt; 2 Department of Animal Reproduction and Artificial Insemination, Veterinary Research Division, National Research Centre, Tahrir St., Dokki 12622, Cairo, Egypt; 3 Department of Animal Health, Animal and Poultry Production Division, Desert Research Center, Cairo, Egypt; 4 Clinic of Obstetrics, Gynecology and Andrology of Large and Small Animals with a Veterinary Ambulance, Justus Liebig University, Giessen, Germany; 5 Department of Physiology, Faculty of Veterinary Medicine, Aswan University, Aswan, Egypt; 6 Department of Theriogenology, Faculty of Veterinary Medicine, Assiut University, 71526 Assiut, Egypt

**Keywords:** data logger, dromedary camels, estrus detection, follicular waves, ovarian hormones, vaginal temperature

## Abstract

**Background and Aim:**

Estrus detection plays a crucial role in the success of animal reproduction. It was previously reported that body temperature changes during estrus. This study aimed to investigate the relationship between vaginal temperatures (VTs) measured by a data logger, ovarian activity, and hormonal cyclic changes in camels.

**Materials and Methods:**

Six mature, healthy, non-pregnant dromedary, and 10-12-year-old camels were included in the study. The ovarian activity was monitored with ultrasonography, and estrus behavior was evaluated using an active and virile male camel. Animals were inserted with a blank controlled internal drug release device attached with an intravaginal data logger. Every hour, the ambient temperature was recorded by another data logger. Blood samples were collected, and sera were used to measure estradiol and progesterone levels.

**Results:**

The whole follicular cycle lasted 25.41±1.36 days, and the maximum sizes of the dominant follicle in the first and second follicular waves were 1.63±0.27 cm and 1.94±0.42 cm, respectively. There was a significant positive correlation between the follicular diameter and estradiol-17b level (p<0.01, r=0.397). There was no correlation between the follicular diameter and progesterone level (p>0.05, r=0.038), which remained low during the whole period of the experiment. The mean daily VT was significantly correlated with the diameter of the dominant follicle (1.7-2.2 cm, p<0.01, r=0.52).

**Conclusion:**

Measurement of VT will improve the accuracy of estrus prediction. Further studies are recommended to validate VT in camel reproduction.

## Introduction

Camels are an important source of nomadic population income in many developing countries of Asia and Africa [1]. Camels are a source of milk, meat, wool, and leather. Camels’ milk is used not only as a nutritious food but also for several therapeutic purposes [2]. Camels are generally reared under extremely harsh conditions concerning temperature, water availability, and nourishment, so their reproductive performance is low [3]. It was reported that male and female camels comply with environmental conditions during the breeding season and sexual activity [4]. There were significant differences in the camel ovarian activity in relation to the months and seasons of the year [4,5]. Camel ovarian activities were recorded between December and May in Egypt [6,7]. During the breeding season, follicular growth constantly occurs in both ovaries in regular waves [8]. Changes in their ovarian follicular dynamics are usually described as a “follicular wave pattern.” The follicular growth was recorded in waves in camels; each one is markedly divided into four phases (recruitment, growth, maturity, and regression) [9,10]. If mating or ovulation-inducing treatment occurs during the mature phase, the follicle ovulates, and a corpus luteum (CL) develops. In the absence of mating or ovulation-inducing treatment, the mature phase is followed by a follicular regression phase, or large anovulatory follicles remain (20-40 mm in diameter) and have free-floating echogenic strands [11]. Estradiol concentration was detected at the highest levels during the mature ovarian phase, and there were strong correlations between estrogen level and follicular size [9,12].

New precision dairy farming technologies allow for continuous monitoring of animal behaviors, which, in turn, can be used to improve herd health and reproductive management [13]. Vaginal temperature (VT) is a good indicator to evaluate the thermoregulatory response in Nellore heifers [14] and dairy cows [15]. Body temperature is related to physiological functions, such as parturition and estrus, in mammals [16,17]. In cows, a decrease in body temperature before parturition ranges from 0.4°C to 1.0°C [16]. Accurate estrus detection and artificial insemination (AI) at the proper time can increase pregnancy rates. Calving interval, milk production, and profitability are affected by the estrous detection of dairy cows [15,18]. In beef cattle, AI is uncommon due to the inadequate estrus detection and effort required for its detection [19]. The recorded VT during estrus increases from 0.3°C to 0.8°C in dairy cattle [15,20]. The increase persists for 7-12 h in dairy cows [20] and buffalo cows [21]. Vaginal skin temperature of sows, gilts, and cows measured using infrared thermography, data logger, or automated VT and activity measuring system, significantly changes during estrus [15,22-24]. In general, assessing the fluctuation of VTs and length of estrus cycles will provide detailed information on accurate estrus detection and determination of the most effective time for mating [25]. No record has related VT to ovarian activity for induced ovulation in *Camelidae* species, including one-humped camels.

This study aimed to determine the relationship between VT measured with data logger and ovarian activity, cycle stages, and steroid hormone concentrations in cyclic dromedary camels.

## Materials and Methods

### Ethical approval

All Institutional and National Guidelines for the care and use of animals were followed according to the Egyptian Medical Research Ethics Committee (no. 14-126).

### Study period and location

The study was carried out in Marsa Matrouh Research Station (latitude, 31°00’ N; longitude, 29°47’ E; temperature, 15.4±3.5°C), Egypt during the breeding season (From November 2018 to February2019),

### Animals

This study was conducted during the breeding season (between November and February) on six adults, non-pregnant, non-lactating, and cyclic *Camelus dromedarius* with an average body weight of 450±21.4 kg and aged 10-12 years. The females were pluripara without any previous puerperal or fertility complications. Camels were housed in an open paddock with a fenced area belonging to the Desert Research Center, Marsa Matrouh research station, Egypt.. The camels were allowed to graze daily from 08:00 H to 14:00 H, and then, Egyptian clover (*Trifolium alexandrinum*) hay was offered *ad libitum*. Freshwater was presented once daily after returning from the pasture. The start of the heat and male receptivity was monitored using an active and virile male camel. A total of 12 non-sequential estrus cycles were investigated during the experiment (two cycles, one without investigation in between, for each animal to decrease the stress).

### Ultrasonographic examination

Before starting and during the experiment, all animals were clinically healthy. The genital tracts of all camels were examined once at the beginning of the experiment with transrectal ultrasonography using a 6/8 MHz linear array transducer connected to a B-mode ultrasound scanner (100 LC, Pie Medical Imaging, Maastricht, Netherlands) and free from any diseases or reproductive disorders. During the examination period, transrectal ultrasonography was performed every 2 days for a maximum of 35 days for each cycle. The females were restrained in sternal recumbence with the four legs properly fastened using ropes. All examinations were conducted by one right-handed operator without using tranquilizers. Throughout the study period, 12 follicular cycles were recorded. At each examination, the number, diameter, and relative position of all follicles ≥4 mm in diameter and luteinized follicle were recorded and sketched on the ovarian charts to analyze the pattern of growth and atresia. When a follicle was not spherical, the mean diameter was obtained as an average of two perpendicular measurements.

### Measurements of environmental and VT

The ambient temperature of the farm was recorded by the data logger every hour (Ondotori Jr., Climatec, Tokyo, Japan). The environmental data logger was placed 1.5 m above the ground to prevent direct sunlight (24 times/day). Using another data logger, the VT was registered every hour (24 times/day) (KN Laboratories, Osaka, Japan). The data logger was attached to a modified progesterone-free vaginal implant device (blank controlled internal drug release device (CIDR, Pfizer, USA). CIDR was inserted vaginally in each animal after cleaning and washing the perineum region with water and povidone iodine-based detergent solution. Besides, 10 mL tetracycline was introduced during the application of CIDR vaginally into each animal. Starting at estrus for each camel, the vaginal data logger was fixed to the CIDR and kept in the vagina for 35 successive days. After the experiment, the data logger was removed, all temperature data (ambient or vaginal) were collected by the software (Rh Manager; KN Laboratories), and the average temperature was calculated by the T&D Recorder Climatec.

### Blood sampling and hormonal analysis

Starting from the day of CIDR insertion, the blood samples were collected through jugular venipuncture every other day. Blood samples were left at room temperature (15.4±3.5°C) to clot, and the sera were harvested by centrifugation at 3000 r.p.m. for 15 min. Sera were kept at −20°C until measurement of estradiol and progesterone levels. Quantitative progesterone and estradiol evaluation was performed using commercial enzyme-linked immunosorbent assay’s enzyme immunoassay kit (Legal Manufacturer, DRG Instruments, GmbH, Germany). The sensitivity was 0.05 ng/mL, and intra-assay and inter-assay precisions were 5.9% and 10.1%, respectively. For estradiol, the sensitivity was 2.0 pg/mL, and intra-assay and inter-assay precisions were 6.81% and 7.25%, respectively.

### Statistical analysis

Data are presented as mean±standard error of the mean. To study the effect of the day during CIDR implantation on mean daily VT, VT at dusk (18:00 H), dawn (06:00 H), noon (12:00 H), and midnight (00:00 H) was determined. Estradiol and progesterone concentrations were studied using a simple one-way analysis of variance (ANOVA). Duncan’s multiple range test was used to differentiate between significant means. To study the effect of the hour during the day on VT, simple one-way ANOVA was used. Pearson correlation coefficient was also performed between follicular diameter, hormones, and mean daily VT and ambient temperature during the same days (all data were pooled for the six females) using SAS software (SAS Inc., NC, USA) [26].

## Results

**Figure-1:**
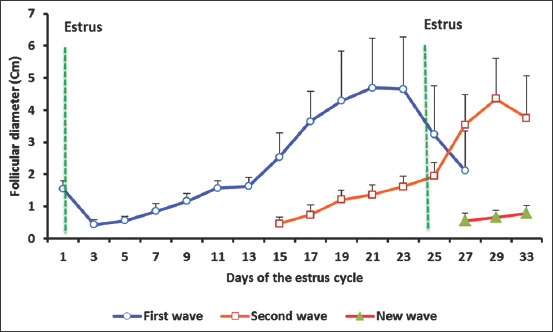
Follicular dynamic during the estrus cycle in camels (n=12, mean±standard error).

The data of follicular measurements are presented in Figure-1. The ovarian activity commenced during the second half of November. The beginning of the ovarian activity was marked by the appearance of the first follicle with a diameter of >4 mm during the time of ultrasonography. Throughout the study period of the follicular dynamics, 12 follicular cycles were recorded. The follicular waves were characterized by three distinct stages: (1) Stage of follicular recruitment: The duration of this phase was 2.9±0.5 days, and this phase was characterized by the presence of multiple small follicles (n=5-10 follicles, 0.4-0.5 cm in diameter). (2) Stage of follicular growth and dominance: Its duration was 8.0±0.3 days, and follicular dominance was established when a follicle emerged from several small follicles of 0.8-0.9 cm in diameter. At the end of this period, the dominant follicle reached the maximum size and remained at the plateau for 5.41±0.6 days. (3) Stage of follicular degeneration: After reaching dominance, the follicle underwent one of two possibilities, regression or development of the cystic structure. The duration of this phase was 9.92±0.31 days. The whole follicular cycle lasted 25.41±1.36 days, and the maximum sizes of the dominant follicle in the first and second follicular waves were 1.63±0.27 cm and 1.94±0.42 cm, respectively.

In 66.7% of the studied cycles (eight cycles), the follicle reached a mature size, whereas, in other 30% of the cycles (four cycles), the follicle continued to grow until it reached a mean diameter of 3.95±0.6 cm (range=30.0-60.0 mm). It was found that both types of follicles were not ovulated in this study without mating. During the regression period, the follicular fluid of these follicles (>2.9 cm in diameter) developed echogenic strands of free-floating fibrin and became more collocated into fibrinous ribbons, with cessation of follicular growth as the follicle degenerated. Nevertheless, these follicles did not inhibit the growth of other follicles in the same or contralateral ovary, and some of them would grow and reached dominance.

**Figure-2:**
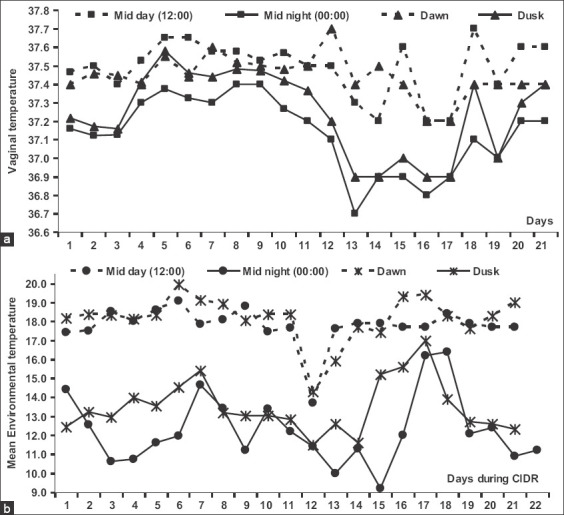
Mean vaginal temperature (a) and environmental temperature (b) in camels recorded with data logger during mid-day (12:00h) and midnight (00:00h), and during dawn (6.00h) and dusk (18.00h).

**Figure-3:**
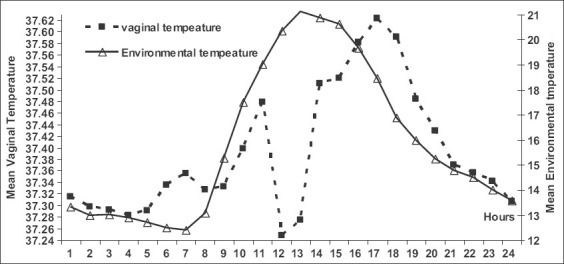
Mean environmental and vaginal temperature/hour after inserting data logger 24 h/day.

The mean daily VT (Figures-2 and 3) was significantly influenced (p=0.0001) by the hour of the day. It was significantly increased at noon (p=0.05) and dawn (p=0.04;) compared to that at midnight and dusk (Figure-2a). In general, the VT at noon (12:00 H, 37.52±0.02°C) was significantly higher (p=0.0001) when compared to at midnight (00:00 H, 37.21±0.03°C) (Figure-2a). The VT recorded at dawn (6:00 H, 37.46±0.02°C) was significantly higher (p<0.0001) when compared to at dusk (18:00 H, 37.26±0.02°C) (Figure-2a). The VT within animals showed significant (p=0.0001) daily cyclic variation. Even though VT increased, all females showed receptivity to males during estrus. The VT of all animals was ≥37.9°C for at least 6 h during the day (Figure-3), where estrus and male receptivity were monitored.

The average ambient temperature recorded with the data logger during the experiment was 15.36°C. The environmental temperature recorded at dawn (06:00 H, 18.51±0.21°C) was significantly higher (p<0.0001) when compared to at dusk (18:00 H, 13.49±0.20°C) (Figure-2b). Moreover, the environmental temperature at noon (12:00 H, 17.98±0.15°C) was significantly higher (p=0.0001) when compared to at midnight (00:00 H, 12.20±0.25°C) (Figure-2b). The daily environmental temperature was also higher at noon and dawn when compared to at midnight and dusk (Figures-2b and 3).

In general, the mean VT (Figure-3) significantly decreased shortly after dusk from 19:00 H till shortly before midnight at 23:00 H and then increased from dawn until it reached its maximum value at noon (12:00 H). The VT was significantly correlated with ambient temperature (r=0.29; p=0.0001).

**Figure-4:**
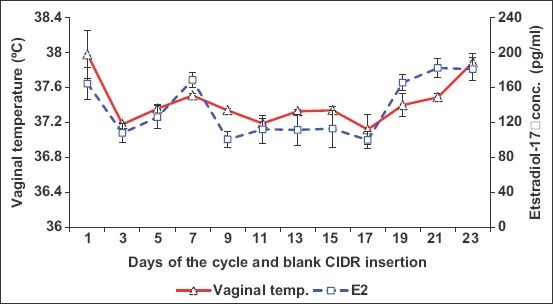
Mean changes in vaginal temperature and estradiol after inserting blank CIDR with data logger intravaginally in cyclic camels.

**Figure-5:**
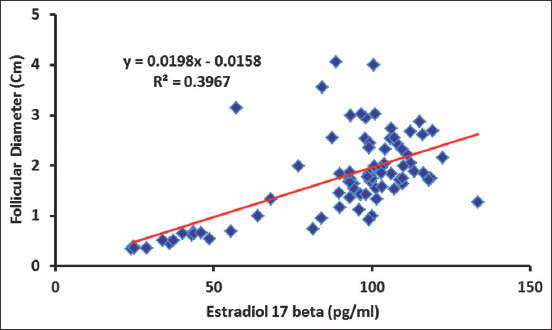
Correlation between the estradiol-17β level (pg/ml) and follicular diameter.

**Figure-6:**
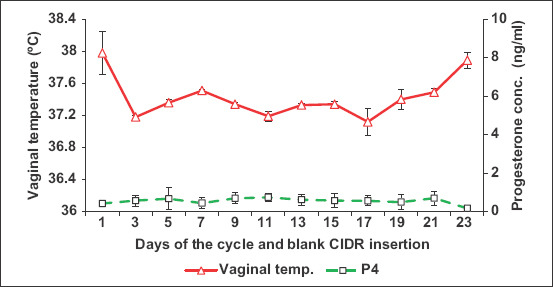
Mean changes in vaginal temperature and progesterone concentration after inserting blank CIDR with data logger intravaginally in cyclic camels.

**Figure-7:**
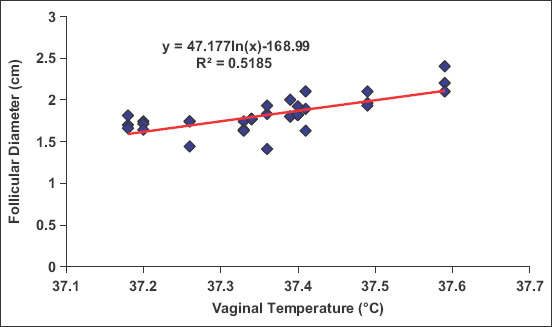
Correlation between the vaginal temperature (°C) and follicular diameter (cm).

**Figure-8:**
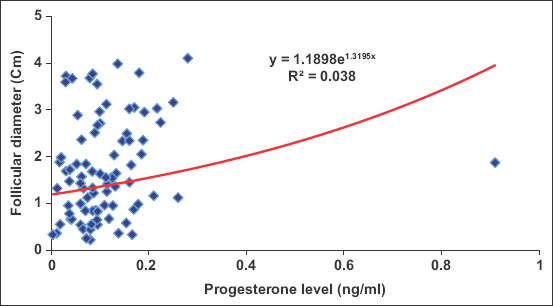
Correlation between the progesterone level (ng/ml) and follicular diameter (cm).

Peripheral serum estradiol concentrations tended to significantly increase (p=0.001) in cyclic camels, reaching its maximum values on days 7 (169.79±42.98 pg/mL), 9 (186.41±28.92 pg/mL), and 13 (281.85±105.34 pg/mL) during estrus cycles with significant variation (p=0.0001) between animals, which is associated with mature follicular (estrus) phase (Figures-4 and 5). The changes in estradiol 17b concentration and VT had strong association (Figure-4). There were no significant changes in the progesterone concentrations throughout the examination periods (Figure-6). There was significant positive correlation between the follicular diameter and estrogen level (p<0.05, r=0.62; Figure-6) and follicular diameter and VT (p<0.01, r=0.72, 1.7-2.2 cm; Figure-7). There was a low positive correlation between VT and progesterone concentration (r=0.19, p<0.05). There was no correlation between the follicular diameter and progesterone level (p>0.05, r=0.038; Figure-8).

## Discussion

In a diversity of domestic animals, the daily rhythmic changes in the body temperature were extensively studied [27]. In Japanese black cows with distinct estrus cycle phases, a daily variation of VT at estrus was related to blood antioxidant levels [22]. Accurate estrous detection could be performed through the continuous measurements of VT and conductivity with a supervised machine in cattle [25]. No record has related VT to ovarian activity for induced ovulation in *Camelidae* species, including one-humped camels. In this study, the VT was ≥37.9ºC on the day where camels showed estrus behavior and male receptivity. The fluctuation in the ruminal temperature of beef cows could be used as a predictor of estrus [28]. Although the ambient temperature was poorly correlated with the VT of dromedary camels, the ambient temperature did not influence the estrus of beef cows [28]. In contrast, in dairy cattle, the ambient temperature affected the body temperature and follicular growth [15,29].

In the current study, the increase in VT persisted at least 6 h during the day; similar findings were recorded in dairy cattle [15,20]. In the current study, the VT recorded with the data logger was consistently high from dawn to dusk when compared to from dusk to midnight, reaching a maximum at 13:00 H and a minimum at midnight. Estrus could be predicted when the VT exceeded 37.5°C at noon. In contrast, cows had higher body temperature at dusk when compared to dawn during three estrus cycles, and body temperature cycle length was similar to estrus cycle length [30]. Rhythmicity of body temperature reflected the 21-day duration of the estrus cycle of cows, and there was no significant seasonal difference during the diestrus period [16,30]. The increased VT of dromedary camels identified in the current study could predict the presence of mature pre-ovulatory follicles. In Bororo zebu cows, body temperature decreased on days 7 and 1 before estrus and 1 day post-estrus [31]. In cows, the VT fluctuated with the estrus cycle, being lowest just before the heat, high on the day of the heat, low again at the time of ovulation, and high during the luteal phase of the cycle [15]. The higher body temperature recorded during this study may relate to the presence of mature dominant follicles and intense activities of camels in estrus. However, body temperature could also be influenced by environmental temperature [32] and season [33]. The marked increase in VT on the day of estrus observed in dromedary camels is also consistent with other animal species [15,22,24]. Our results on follicular wave presented great similarities with those found in llamas and alpacas [34] and camels [9,10], where they postulated that the mechanisms of recruitment of each follicular wave are caused by an increase in follicle-stimulating hormone.

The strong correlation between estradiol and VT, estrus behavior, and receptivity to male dromedary camel of the present study are similar to the increased plasma estradiol concentrations for 12 days after the beginning of the growth phase when the diameter of the largest dominant follicle reached 18.7 mm [35], and the presence of a strong correlation between the concentration of estradiol, estrus activity, and VT during estrus in dairy cows [20]. The increase in estrogen-stimulated behavioral estrus was noted during the mature follicular phase, in which the female becomes receptive to mating and exhibits estrus behavioral patterns [15]. The external signs of heat observed in dromedary camels were less evident than that in cows and mares [9,36]. However, it was reported that camels showed intense estrus behavior and receptivity to the male with high estrogen levels during the days of the mature follicular phase [37].

The progesterone levels exhibited no variations among the examined camels throughout the examination periods. These results agree with previously reported results, where it was found that the plasma progesterone level in the non-pregnant female camels is extremely low [37]. In contrast, spontaneous ovulation increased progesterone for 7-9 days [35], or CIDR-induced ovulation, which was observed in camels carrying progesterone releasing intravaginal devices [36]. Otherwise, the presence of luteinized pre-ovulatory follicle at the time of CIDR insertion to dromedaries produced progesterone concentrations similar to the CL [38]. It was reported that a strong relationship exists between rectal and VT. Furthermore, the VT may provide a more sensitive and reliable estimate of the core body temperature than the rectal one in grazing *Bos taurus* heifers [39] and dairy cattle [15].

## Conclusion

The VT can be used as a marker for predicting estrus or ovulation in camels. Environmental temperature has little effect on VT. The VT decreased from dusk and reached the minimum value at midnight and increased from 06:00 H to 14:00 H when the VT could be measured during these hours.

## Authors’ Contributions

RM, AA, RSM, AW, FA, and HAH: Carried out the main research works. RM, AA, AW, and HAH: Designed the study. RM, RSM, and HAH: Data collection. AA and FA: Analysis, and interpretation of the data. HAH and AW: Drafted the manuscript. All authors read and approved the final manuscript.
